# Assessment of bite pressure differences between implant-supported prostheses and natural dentition

**DOI:** 10.6026/973206300212581

**Published:** 2025-08-31

**Authors:** Sushrima Dey, Soumya Iranna Dudhani, Deesha Chhaya, Sapam Shinttoo Devi, Monesh Shashikant Sharma, Rahul Raviraj Shetty, Miral Mehta

**Affiliations:** 1 Department of Periodontology & Oral Implantology, Kalinga Institute of Dental Sciences, KIIT Deemed to be University Bhubaneswar, Odisha, India; 2 Department of Prosthodontics, Crown & Bridge, Government Dental College and Hospital, Jamnagar, Gujarat, India; 3 Department of Periodontics and Oral Implantology, College of Dental Sciences and Research Center, Ahmedabad, Gujarat, India; 4 Department of Prosthodontics, Crown and Bridge, Teerthankeer Mahaveer Dental College and Research Centre, Moradabad (TMU), Uttar Pradesh, India; 5 Department of Prosthodontics and Crown and Bridge, SMBT Institute of Dental Sciences and Research, Dhamangaon, Maharashtra, India; 6 Department of Oral Medicine and Radiology, Bharati Vidyapeeth(Deemed to be University)Dental College and Hospital, Navi Mumbai, India; 7 Department of Pediatric and Preventive Dentistry, Karnavati School of Dentistry, Karnavati University, Gandhinagar, Gujarat, India

**Keywords:** Bite force, dental implants, prosthetic rehabilitation, occlusal analysis, digital dentistry

## Abstract

A comparative analysis of the bite pressure between the mandibular first molars region using implant-supported prostheses and the contralateral side natural teeth is of interest. NUPAI and T-Scan III systems were used in the assessment of thirty-five patients. Data shows that maximum (34.2 vs 28.7 MPa) and average bite pressures (25.8 vs 21.4 MPa) and their contact areas (28.4 vs 24.1 mm 2) were noticeably greater on the natural tooth side (p < 0.05). The males showed an 18 percent higher bite power and differences were observed with age. Thus, natural teeth are better than implants because of proprioceptive feedback, which justifies the importance of appropriate adjustment of occlusion in implant prosthodontics.

## Background:

Remodelling of masticatory capacity using dental implant treatment has assumed a keystone feature of contemporary restorative dental care and the success achieved has been more than 95 percent after 10 years [1]. There are, however, basic biomechanical disparities between natural teeth and osseointegrated implants that can affect the occlusal dynamics and production of bite forces [2, 3]. Being aware of these differences is vital in the achievement of the best results as well as to avoid complications that may arise out of the process, like implant overloads and prosthetic failures. Human teeth have a complicated proprioceptive system dependent on periodontal ligament mechanoreceptors that supply the sensory feedback in the course of mastication [4]. Such a neurosensory system, with its ability to modulate force, allows accurate force control and guards against over-occlusal loads [5]. Conversely, osseointegrated implants do not imply periodontal ligaments and functions are inserted with different theatrics defined as "osseoperception" [6]. Such fundamental disparity can lead to a changed bite force pattern and impaired tactile perception, focusing on implant-retained restoration. In the above studies, it has been shown that dental implant patients are capable of producing forces between 50 N and 900 N, with the posterior regions showing the ability of forces x3 in comparison with the anterior region [7]. Comparative studies of implant-supported prostheses to natural dentition have produced variable findings, with some findings similar to bite forces and others a dramatic decrease in bite forces and resulting in implant-supported restorations [8, 9]. After applying implants, it could be overloaded because of poor proprioceptive feedback, which would result in loss of bone and subsequent failure of the fixtures [10]. Therefore, it is of interest to assess the bite pressure differences between implant-supported prostheses and natural dentition.

## Materials and Methods:

Participants included adults aged 35 to 65 years who presented with a unilateral implant-supported crown in the mandibular first molar region and had an intact contralateral natural mandibular first molar. All included implants were functionally loaded for at least six months, and participants exhibited stable occlusion with no clinical signs of temporomandibular disorders. Complete healing of peri-implant tissues was also a prerequisite. Exclusion criteria encompassed the presence of active periodontal disease or peri-implantitis, bruxism or other parafunctional habits, neurological disorders affecting masticatory function, medication use known to influence muscle activity, incomplete osseointegration, and any history of prosthetic complications or repairs. Sample size estimation was conducted using G*Power version 3.1.9.7. Assuming an effect size of 0.8, an alpha error of 0.05, and a power of 90%, the minimum required sample size was calculated to be 32 participants. To compensate for potential dropouts, 35 participants were ultimately recruited. Bite pressure measurements were carried out using two complementary systems: the NUPAI Bite Scan System (Novel GmbH, Munich, Germany) for pressure-sensitive film analysis and the T-Scan III Novus Digital Occlusal Analyzer (Tekscan Inc., South Boston, MA, USA) for real-time digital force evaluation. Participants were seated in a standardized dental chair with the Frankfurt horizontal plane aligned parallel to the floor. A five-minute acclimatization period was allowed to ensure muscle relaxation. The measurement protocol included a calibration phase in which each system was adjusted per the manufacturer's specifications, followed by proper head positioning using a stabilizing headrest to maintain consistent mandibular relationships. Sensors were placed bilaterally at the first molar contact points using both the pressure-sensitive films and the T-Scan sensors. Participants were instructed to perform maximum voluntary clenching for three seconds, with a rest interval of 60 seconds between each attempt. Three separate recordings were made on each side, and the highest value obtained was used for final analysis. The key parameters collected for each measurement site included maximum bite pressure (MPa), average bite pressure (MPa), contact area (mm^2^), force distribution patterns, and time to reach maximum force (ms). Data were analyzed using SPSS version 28.0 (IBM Corporation, Armonk, NY, USA). Descriptive statistics, including means, standard deviations, and 95% confidence intervals, were calculated. Normality of the data distribution was assessed using the Shapiro-Wilk test. Paired t-tests were used to compare the bite pressure parameters between implant-supported and natural tooth sites. Gender-based comparisons were analyzed using independent t-tests, and age group differences were examined through one-way ANOVA. Pearson correlation coefficients were calculated to assess relationships between variables. A p-value of less than 0.05 was considered statistically significant.

## Results:

Thirty-five participants completed the study protocol (18 males, 17 females; mean age 52.4 ± 8.7 years, range 37-64 years). All implants (Nobel Biocare Replace Select, Nobel Biocare AB, Göteborg, Sweden) demonstrated successful osseointegration with a mean functional loading time of 14.2 ± 6.8 months. Demographic characteristics are presented in Table 1 (see PDF). Significant differences were observed between natural teeth and implant-supported prostheses across all measured parameters. Maximum bite pressure on natural teeth (34.2 ± 4.1 MPa) was significantly higher than implant prostheses (28.7 ± 3.8 MPa) (p < 0.001, 95% CI: 4.2-6.8 MPa). This represents a 19.1% reduction in maximum bite pressure for implant-supported restorations compared to natural teeth. Average bite pressure demonstrated similar patterns, with natural teeth generating 25.8 ± 2.9 MPa compared to 21.4 ± 2.7 MPa for implant prostheses (p < 0.001, 95% CI: 3.1-5.7 MPa). The mean difference of 4.4 MPa represents a 17.1% reduction in average bite pressure for implant sites. Contact area measurements revealed larger surface engagement for natural teeth (28.4 ± 5.2 mm^2^) versus implant prostheses (24.1 ± 4.6 mm^2^) (p = 0.003, 95% CI: 1.5-6.1 mm^2^). The reduced contact area for implant prostheses may contribute to concentrated stress distribution and altered force transmission patterns (Table 2 - see PDF). Male participants demonstrated significantly higher bite pressures compared to females across both restoration types. For natural teeth, males generated 37.1 ± 3.8 MPa versus females at 31.0 ± 3.2 MPa (p < 0.001). Similar patterns were observed for implant prostheses, with males producing 31.2 ± 3.4 MPa compared to females at 25.9 ± 2.9 MPa (p < 0.001). This represents an 18% gender-related difference in bite force generation. Participants were stratified into three age groups: 35-45 years (n=11), 46-55 years (n=13), and 56-65 years (n=11). ANOVA revealed significant age-related decreases in bite pressure for both restoration types (p = 0.007). Post-hoc analysis showed that the youngest group (35-45 years) generated significantly higher forces than the oldest group (56-65 years) for both natural teeth and implant prostheses. Strong positive correlations were identified between natural tooth and implant prosthesis bite pressures within individual participants (r = 0.742, p < 0.001), suggesting that patient-specific factors significantly influence force generation capacity. Body mass index demonstrated moderate positive correlation with bite force values (r = 0.523, p = 0.001), while age showed negative correlation (r = -0.401, p = 0.017). Analysis of force development patterns revealed that natural teeth achieved maximum bite pressure faster (485 ± 89 ms) compared to implant prostheses (523 ± 102 ms) (p = 0.012). This 38-millisecond delay in force development for implants may reflect reduced neurosensory feedback and altered motor control patterns.

## Discussion:

This study provides comprehensive quantitative evidence demonstrating superior bite pressure generation in natural teeth compared to implant-supported prostheses within the same individuals. The 19.1% reduction in maximum bite pressure for implant restorations aligns with previous research reporting functional differences between natural and artificial tooth replacement systems [11]. The observed differences in bite pressure can be attributed to fundamental biomechanical distinctions between natural teeth and osseointegrated implants. Natural teeth benefit from periodontal ligament mechanoreceptors that provide continuous proprioceptive feedback during mastication [12]. These mechanoreceptors enable precise force modulation and contribute to the protective reflexes that prevent excessive loading [13]. The absence of these receptors in implant-supported restorations results in reduced tactile sensitivity and altered neuromuscular control patterns. The concept of "osseoperception" has been proposed to explain compensatory mechanisms in implant-supported restorations [14]. While osseointegrated implants develop alternative sensory pathways through bone, periosteal and muscle receptors, these mechanisms appear insufficient to fully replicate the sophisticated feedback systems of natural teeth [15]. Our findings of delayed force development in implant prostheses (523 ms vs 485 ms) support this hypothesis and suggest compromised neuromuscular coordination. The reduced bite pressure capacity of implant prostheses has important clinical implications for treatment planning and prosthetic design. The 17.1% reduction in average bite pressure may necessitate modified occlusal schemes to optimize force distribution and prevent overloading of natural teeth during bilateral function [16]. Current findings support previous recommendations for implementing "implant-protected occlusion" concepts that minimize lateral forces on osseointegrated fixtures [17]. The observed reduction in contact area for implant prostheses (24.1 mm^2^ vs 28.4 mm^2^) may contribute to concentrated stress patterns and increased risk of prosthetic complications. This finding emphasizes the importance of optimizing crown morphology and contact point design to maximize functional surface area and improve force distribution characteristics [18]. Our results are consistent with recent studies using similar measurement methodologies. A comparative assessment by Geckili *et al.* reported average pressures of 25.33 MPa for natural teeth versus 21.27 MPa for implant prostheses, closely matching our findings of 25.8 MPa and 21.4 MPa, respectively [19]. The consistent results across different populations and measurement systems strengthen the validity of observed differences [20]. However, their research focused on edentulous patients receiving implant-supported overdentures rather than single-tooth replacements, limiting direct comparison with our single-crown results. The 18% higher bite forces in male participants align with established literature documenting gender-related differences in masticatory muscle strength [21]. These differences appear consistent across both natural teeth and implant prostheses, suggesting that patient-specific factors influence overall force generation capacity regardless of restoration type. Age-related reductions in bite pressure reflect natural changes in muscle mass, bone density and neuromuscular coordination that occur with aging [22]. The strong correlation between natural tooth and implant prosthesis forces within individuals (r = 0.742) indicates that patient-specific physiological factors significantly influence outcomes for both restoration types. Several limitations should be acknowledged in interpreting these results. The cross-sectional design provides snapshot data that may not reflect long-term functional adaptations. The study focused exclusively on mandibular first molar replacements, and results may not be generalizable to other anatomical locations or implant configurations. Additionally, the measurement protocol utilized maximum voluntary clenching, which may not accurately represent functional chewing forces during normal mastication [23]. The relatively short mean loading time (14.2 months) may not allow for complete neural adaptation and osseoperception development. Longitudinal studies examining bite pressure changes over extended periods would provide valuable insights into adaptive mechanisms and long-term functional outcomes [24]. Future investigations should examine bite pressure patterns during functional activities such as chewing specific food textures rather than maximum voluntary clenching. Advanced technologies, including electromyography and kinematic analysis, could provide deeper insights into neuromuscular adaptations following implant treatment. The development of intelligent bite force monitoring systems using MEMS pressure sensors, as proposed by recent research, may enable real-time feedback and prevent implant overloading [16]. Such technologies could revolutionize implant dentistry by providing continuous monitoring and patient education regarding appropriate force levels.

## Conclusion:

Natural teeth produce significantly higher bite pressures than implant-supported prostheses due to biomechanical and proprioceptive differences. Reduced contact area, delayed force development, and patient-specific factors highlight the need for optimized occlusal design and implant-protective protocols. Future research should explore long-term functional adaptations and smart monitoring technologies to improve implant outcomes.
